# Deep learning in forensic gunshot wound interpretation—a proof-of-concept study

**DOI:** 10.1007/s00414-021-02566-3

**Published:** 2021-04-06

**Authors:** Petteri Oura, Alina Junno, Juho-Antti Junno

**Affiliations:** 1 Center for Life Course Health Research, Faculty of Medicine, University of Oulu, Oulu, Finland; 2 Cancer and Translational Medicine Research Unit, University of Oulu, Oulu, Finland; 3 Department of Archaeology, Faculty of Humanities, University of Oulu, Oulu, Finland; 4 Faculty of Arts, University of Helsinki, Helsinki, Finland

**Keywords:** Forensic medicine, Gunshot, Wound, Artificial intelligence, Deep learning, Piglet carcass

## Abstract

While the applications of deep learning are considered revolutionary within several medical specialties, forensic applications have been scarce despite the visual nature of the field. For example, a forensic pathologist may benefit from deep learning-based tools in gunshot wound interpretation. This proof-of-concept study aimed to test the hypothesis that trained neural network architectures have potential to predict shooting distance class on the basis of a simple photograph of the gunshot wound. A dataset of 204 gunshot wound images (60 negative controls, 50 contact shots, 49 close-range shots, and 45 distant shots) was constructed on the basis of nineteen piglet carcasses fired with a .22 Long Rifle pistol. The dataset was used to train, validate, and test the ability of neural net architectures to correctly classify images on the basis of shooting distance. Deep learning was performed using the AIDeveloper open-source software. Of the explored neural network architectures, a trained multilayer perceptron based model (MLP_24_16_24) reached the highest testing accuracy of 98%. Of the testing set, the trained model was able to correctly classify all negative controls, contact shots, and close-range shots, whereas one distant shot was misclassified. Our study clearly demonstrated that in the future, forensic pathologists may benefit from deep learning-based tools in gunshot wound interpretation. With these data, we seek to provide an initial impetus for larger-scale research on deep learning approaches in forensic wound interpretation.

**Supplementary Information:**

The online version contains supplementary material available at 10.1007/s00414-021-02566-3.

## Introduction

Artificial intelligence uses trained algorithms to mimic human cognitive functions [1], especially in the context of interpreting complex data [2]. In contrast to conventional methods, artificial intelligence algorithms are allowed to approach problems freely without strict programming [3]. Deep learning is a subcategory under artificial intelligence, utilizing neural networks in a wide range of concepts such as image, text, and speech recognition [2, 4, 5]. While the applications of artificial intelligence and deep learning techniques are considered revolutionary within the healthcare sector and several medical specialties [2, 4, 6, 7], forensic applications have been relatively scarce [3, 8–11] and centered on subfields other than forensic pathology. This somewhat surprising, given the visual nature of forensic pathology at both microscopic and macroscopic levels.

The vast majority of missile wounds are caused by firearms [12]. In the USA, more than 30,000 people sustained lethal gunshot trauma in 2014, roughly two-thirds being suicides and one-third homicides [13]. As a thorough investigation of the course of events is in the public interest with potentially far-reaching legal consequences, the forensic pathologist’s conclusion of events should naturally be as accurate as possible. However, in case of lacking background information (in terms of, e.g., weapon type, bullet type, gunshot trajectory, and shooting distance [12, 14, 15]), these need to be inferred solely on the basis of the wounds and injuries detected by examining the victim’s body. To the best of our knowledge, there are no previous studies addressing the potential of deep learning in assisting the forensic pathologist in these cases.

In our proof-of-concept study, we aimed to test the hypothesis that deep learning algorithms have potential to predict shooting distance class out of four possibilities on the basis of a simple photograph of a gunshot wound. Due to the preliminary nature of our study, we utilized nineteen piglet carcasses as our material. A dataset of 204 images of gunshot wounds (60 negative controls, 50 contact shots, 49 close-range shots, and 45 distant shots) were used to train, validate, and test the ability of neural net architectures to correctly classify images on the basis of shooting distance. With these data, we sought to provide an initial impetus for larger-scale research on deep learning approaches to forensic wound interpretation.

## Materials and methods

### Material

This study tested the potential of deep learning methods to predict shooting distance class on the basis of a photograph of the gunshot wound. Nineteen carcasses of freshly died farmed piglets (weight range 2.05–4.76 kg) were used as the study material. The piglets had all sustained a natural death and were stored in a cold restricted room for ≤ 5 days until collected for this experiment. Exclusion criteria included external deformity and abnormal or blotchy skin pigmentation. Pig and piglet carcasses have been frequently and successfully used in studies on taphonomic processes and other forensic purposes [16]. While pigs do not perfectly act as a substitute for humans, they were chosen for this proof-of-concept study in order to avoid the obvious ethical concerns related to the use of human cadavers and minimize the risk of sensationalism. Importantly, pigs have rather similar skin attributes to humans [16, 17].

This study did not involve laboratory animals, living animals, or human cadavers. No piglets were harmed for this experiment, as all of them had died naturally in young age and were collected for this study afterwards. The carcasses were delivered for disposal according to local regulations immediately after the experiment.

### Infliction of gunshot wounds

The .22 Long Rifle (5.6 × 15 mm) is a popular rimfire caliber in rifles and pistols. Contrary to general view as a low-powered and less dangerous caliber, .22 Long Rifle firearms are often used in shootings and homicides [18]. For example in Australia 43% [19] and in New Zealand 33% [20] of firearm homicides were committed using a .22 caliber rimfire. For this study, we selected the Ruger Standard Model semiautomatic pistol which is one of the most popular rimfire pistols globally. A 5 1/2 barrel length version of Mark II model was used in our experiment.

We selected to use the pistol version of .22 Long Rifle ammunition as we assumed that the powder type and amount are optimized with the shorter barrel length of pistols. We used Lapua Pistol King cartridge with 2.59 g (40 grain) round nose unjacketed lead bullet. Reported muzzle velocity with the barrel length of 120 mm is 270 m/s.

**Fig. 1:**
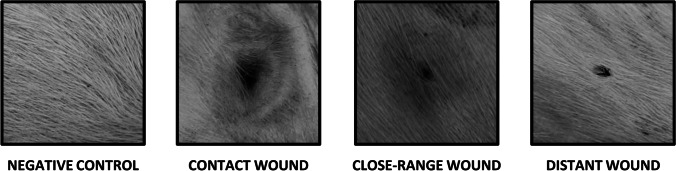
Representative examples of wound types

For this proof-of-concept study, we restricted shooting distances to three discrete classes, namely contact shot (0 cm), close-range shot (20 cm), and distant shot (100 cm). These were selected on the basis of previous literature regarding gunshot wound interpretation and classification among humans [12, 14, 15]. Fifty rounds of each distance were fired, with a grand total of 150 rounds in our experiment. Examples of typical wound appearances are presented in Fig. 1.

### Photography of gunshot wounds

A professional 24.2-megapixel digital single-lens reflex Canon EOS 77D camera with Canon EF-S 17–55 mm f/2.8 IS USM lens (Canon, Tokyo, Japan) were used to obtain photographs of the entry wounds. Photographs were taken in a 90-degree angle towards the carcass, after ensuring an even lighting.

### Experimental setting

As the experiment involved a firearm, safety measures were carefully planned beforehand. The experiment was performed in a restricted area, and only researchers whose presence was essential were present during the experiment. A researcher with a valid firearms license and long-term experience with firearms, including the one used in this study, performed the experiments (JAJ).

Due to the preliminary nature of the study, we aimed to keep all circumstantial factors fixed, except for shooting distance. First, a carcass was visually inspected and positioned on its left/right side onto a sandbed with a paperboard between the carcass and the sandbed. A set of clean photographs (negative controls) were obtained. Then, a round of 5–10 shots (depending on the size of the carcass) was fired vertically in a 90-degree angle at its upward-facing side, excluding face and distal limbs. Each carcass was randomized to sustain either contact shots, close-range shots, or distant shots using a random number generator (SPSS Statistics version 26, IBM, Armonk, NY, USA). An adjustable ruler was used to ensure the accuracy of shooting distance. Lastly, the carcass was photographed. The external circumstances such as weather and lighting conditions remained unchanged during the course of the experiment.

### Image curation and post-processing

At the post-experiment phase, the full-sized photographs obtained during the experiment were uploaded to Photoshop Elements version 2021 (Adobe, San Jose, CA, USA) for visual inspection. Gunshot wounds (*n* = 144) were identified and extracted using a 420 × 420-pixel square cutter in .png format. Negative controls (*n* = 60) were extracted from photographs with no gunshot wounds following an identical method, with the exception that the extracted regions were chosen at random, without allowing two regions to overlap. The final dataset comprised a total of 204 images. Examples of extracted images are presented in Fig. 1.

Using a random number generator, the images of the final dataset were randomly divided into three sets (i.e., training, validation, and test sets) to be utilized in the deep learning procedure. In order to ensure sufficient set sizes, we used a 60%-20%-20% ratio in the division. Each of the three sets contained roughly equal percentages of negative controls, contact shots, close-range shots, and distant shots (Table 1).

**Table 1:** Division of data into training, validation, and test sets

	Training set	Validation set	Test set
Size (% of total)	122 (59.8%)	41 (20.1%)	41 (20.1%)
Class 1 (negative controls)	36	12	12
Class 2 (contact wounds)	30	10	10
Class 3 (close-range wounds)	29	10	10
Class 4 (distant wounds)	27	9	9

### Deep learning procedure

Deep learning was performed using the open-source AIDeveloper software version 0.1.2 [5] which has a variety of neural net architectures readily available for image classification. The curated images were uploaded to AIDeveloper as 32 × 32-pixel grayscale images in order to streamline the deep learning process due to the relatively small sample size.

First, we used the training and validation image sets to train models (up to 3000 epochs) by means of all the available architectures. The specific parameters used in AIDeveloper are presented in Supplementary Table 1. Based on the validation accuracies obtained during the training process, we selected the best-performing model within each architecture. The best-performing models were then tested using the test set; performance metrics of all trained models are presented in Supplementary Table 2. In the results section, we present the model with the highest performance metrics from the test set round.

We assessed the models on the basis of the following metrics: testing accuracy (i.e., (true positive + true negative)/all), precision (i.e., true positive/(true positive + false positive)), recall (i.e., true positive/(true positive + false negative)), F1 value (i.e., 2 x (precision x recall)/(precision + recall)), and area under the receiver operating characteristics curve (i.e., a measure of the performance of a test relative to the ideal where there are no false negative or false positive results) [5, 21]. We did not aim to publish the algorithm of the best-performing model, as its applicability among other populations (e.g., external piglet carcasses and human cadavers) was likely to be poor. However, the algorithms are available from the corresponding author on request.

## Results

Of the explored neural network architectures, a trained multilayer perceptron based model (MLP_24_16_24) reached the highest testing accuracy of 98%. The components of this model are specified in Table 2. A confusion matrix (i.e., a contingency table where predicted class is plotted against true class) and performance metrics for each class are presented in Tables 3 and 4, respectively. Of the testing set, the trained model was able to correctly classify all negative controls, contact shots, and close-range shots, whereas one distant shot was misclassified.

**Table 2:** Neural net architecture of the best-performing multilayer perceptron model (MLP_24_16_24)

Layer number	Component
1	Input
2	Dense *n* = 24
3	Rectified linear unit
4	Dense *n* = 16
5	Rectified linear unit
6	Dense *n* = 24
7	Rectified linear unit
8	Output

**Table 3:** Confusion matrix demonstrating true and predicted classes on test set data (bold signifies correct predictions)

	Predicted class			
	Class 1	Class 2	Class 3	Class 4
True class				
Class 1	**100.0% (12/12)**	0.0% (0/12)	0.0% (0/12)	0.0% (0/12)
Class 2	0.0% (0/10)	**100.0% (10/10)**	0.0% (0/10)	0.0% (0/10)
Class 3	0.0% (0/10)	0.0% (0/10)	**100.0% (10/10)**	0.0% (0/10)
Class 4	11.1% (1/9)	0.0% (0/9)	0.0% (0/9)	**88.9% (8/9)**

**Table 4:** Performance metrics of the trained neural network model against the test set

Metric	Class 1	Class 2	Class 3	Class 4
Testing accuracy	0.96	1.00	1.00	0.94
F1	0.96	1.00	1.00	0.94
Precision	0.92	1.00	1.00	1.00
Recall	1.00	1.00	1.00	0.89
Area under the receiver operating characteristics curve	0.99	1.00	1.00	0.99

## Discussion

In this proof-of-concept study, we aimed to study the ability of deep learning methods to correctly estimate shooting distance on the basis of gunshot wound photographs. In our dataset of 204 images, a trained multilayer perceptron based neural net architecture proved most accurate, reaching the highest testing accuracy of 98%. As such, our results encourage larger-scale research on deep learning approaches to forensic wound interpretation.

Although artificial intelligence and deep learning techniques have clinical applications in several medical specialties [2, 4, 6, 7], forensic applications have been relatively scarce [3, 8–11] and centered on subfields other than forensic pathology. To the best of our knowledge, this is the first study to address deep learning in gunshot wound interpretation. The present dataset comprised images from four discrete classes, namely contact shot, close-range shot, and distant shot wounds, as well as negative controls with no wounds. Each of the wound types was considered to have distinct visual features, thus providing a basis for the deep learning approach. Of the independent testing set, the fully trained multilayer perceptron based model was able to correctly classify all negative controls (100%), contact shots (100%), and close-range shots (100%) and misclassified one distant shot as a negative control (88.9%). Even though the division into four classes was relatively rough, the present results suggest that forensic pathologists may benefit from deep learning algorithms in gunshot wound interpretation.

The main limitations of the study include a relatively small sample comprised of non-human material (204 photographs from 19 piglet carcasses) and the focus on only four gunshot wound classes. While only one weapon type and caliber were used in this preliminary study, further studies are needed to challenge the deep learning process with higher levels of variation in the dataset (e.g., weapon type, bullet type, shot trajectory, and circumstantial factors). Apart from the training, validation, and test sets needed for the training and testing of the algorithms, this study had no external validation set. Furthermore, we did not compare the performance of the algorithm to the gold standard (i.e., an experienced forensic pathologist). Therefore, the applicability of the present algorithm is highly limited in external settings. However, given the proof-of-concept nature and the high classification accuracy of the best-performing algorithm reached in this study, it seems clear that future research is needed to develop more robust and widely applicable algorithms for forensic use.

Future studies are encouraged to develop robust algorithms using large image sets of human material from diverse sources. In large datasets, shooting distance should be modelled continuously instead of discrete classes in order to increase the applicability of the estimates. Alongside shooting distance, algorithms should also be developed for other relevant indices such as weapon type, bullet type, and gunshot trajectory [12, 14, 15], or in a wider context also for other missile wounds, blunt trauma, or virtually any lesion with forensic significance. Ideally, prospective multicenter image libraries of substantial magnitude would offer outstanding material for the systematic development of accurate and scientifically validated algorithms.

From the legal perspective, the use of artificial intelligence to generate robust and comprehensible evidence remains problematic. Although artificial intelligence may be subject to human-like bias through the way it processes datasets and assesses events, it is difficult to examine similarly to human witnesses in the courtroom [22]. However, we hope that deep learning algorithms will, in the future, provide forensic pathologists with tools to utilize when constructing a view of the course of events. For example, the tools may assist in screening complex datasets for a specific detail or highlight areas of potential interest to the forensic pathologist. Importantly, the final interpretation would always remain with the forensic pathologist.

In this proof-of-concept study, a trained deep learning algorithm was able to predict shooting distance class (negative control, contact shot, close-range shot, or distant shot) on the basis of a simple photograph of a gunshot wound with an overall accuracy of 98%. The results of this study encourage larger-scale research on deep learning approaches to forensic wound interpretation.

## Supplementary Information

Below is the link to the electronic supplementary material.

Supplementary file1 (DOCX 20 KB)

## Data Availability

The datasets and algorithms generated and analyzed during the study are available from the corresponding author on request.
