# Supplementary material for: Deep learning in forensic gunshot wound interpretation—a proof-of-concept study
Source: Int J Legal Med. 2021 Apr 6;135(5):2101–6. doi: 10.1007/s00414-021-02566-3 (PMC8354947; doi:10.1007/s00414-021-02566-3)
Supplement: Supplementary file 1 — Supplementary file1 (DOCX 20 KB) [file 414_2021_2566_MOESM1_ESM.docx]

**SUPPLEMENTARY TABLES**

Title

DEEP LEARNING IN FORENSIC GUNSHOT WOUND INTERPRETATION – A PROOF-OF-CONCEPT STUDY

Author List

Petteri Oura^1^, MD, PhD
Alina Junno^2,3^
Juho-Antti Junno^2,3,4^, PhD

Author Affiliations

1. Center for Life Course Health Research, Faculty of Medicine, University of Oulu, Oulu, Finland.
2. Cancer and Translational Medicine Research Unit, University of Oulu, Oulu, Finland.
3. Department of Archaeology, Faculty of Humanities, University of Oulu, Oulu, Finland.
4. Archaeology, Faculty of Arts, University of Helsinki, Helsinki, Finland.

**Supplementary Table 1**. Parameters of the neural network training process in AIDeveloper.

| Parameter | Value |
| --- | --- |
| Model specification parameters |  |
| Network architecture | Several tested, please refer to **Supplementary Table 2**. |
| Input image size (pixels) | 32 x 32 |
| Image normalization | Division by 255 |
| Color mode | Grayscale |
| Padding | No |
| Total number of epochs | 3000 |
| Image augmentation parameters, set I |  |
| Vertical flip | Yes |
| Rotation (degrees, range) | -3…3 |
| Width shift (%, range) | -0.1…0.1 |
| Height shift (%, range) | -0.1…0.1 |
| Zoom (%, range) | -0.1…0.1 |
| Shear (%, range) | -0.5…0.5 |
| Number of epochs after which refreshes | 2 |
| Image augmentation parameters, set II |  |
| Brightness by addition (%, range) | -15…15 |
| Brightness by multiplication (%, range) | 0.7…1.3 |
| Contrast (%, range) | 0.7…1.3 |
| Gaussian noise (mean with standard deviation) | 0.0 (3.0) |
| Blurring (kernel size, range) | 0…5 |
| Number of epochs after which refreshes | 1 |

**Supplementary Table 2**. Performance metrics of the explored neural network models.

| Network architecture^1^ | Train and validation set | | |  | Test set | |
| --- | --- | --- | --- | --- | --- | --- |
|  | Best epoch | Training accuracy | Validation accuracy |  | Testing accuracy | Correct per class (%) |
| **MLP_24_16_24** | **1494** | **0.95** | **1.00** |  | **0.98** | **100.0/100.0/100.0/88.9** |
| MLP_64_80_32 | 566 | 0.93 | 0.98 |  | 0.93 | 100.0/100.0/90.0/77.8 |
| LeNet5 | 117 | 0.90 | 1.00 |  | 0.93 | 100.0/100.0/80.0/88.9 |
| LeNet5_do | 174 | 0.91 | 1.00 |  | 0.93 | 100.0/100.0/80.0/88.9 |
| MLP_24_16_24_skipcon | 979 | 0.95 | 0.98 |  | 0.90 | 100.0/100.0/80.0/77.8 |
| LeNet5_bn_do_skipcon | 318 | 0.98 | 0.98 |  | 0.90 | 100.0/100.0/70.0/88.9 |
| MLP_8_8_8 | 471 | 0.82 | 0.88 |  | 0.88 | 100.0/100.0/90.0/55.6 |
| MLP_64_32_16 | 745 | 0.95 | 0.98 |  | 0.88 | 100.0/100.0/70.0/77.8 |
| MLP_72_48_24_32 | 1009 | 0.96 | 0.98 |  | 0.88 | 100.0/100.0/70.0/77.8 |
| MLP_72_64_48_48 | 793 | 0.97 | 0.98 |  | 0.88 | 100.0/100.0/70.0/77.8 |
| LeNet5_bn_do | 1193 | 0.99 | 1.00 |  | 0.88 | 100.0/80.0/70.0/100.0 |
| TinyCNN | 2514 | 0.99 | 1.00 |  | 0.88 | 100.0/100.0/60.0/88.9 |
| MLP_16_8_16 | 2515 | 0.89 | 0.88 |  | 0.85 | 100.0/100.0/80.0/55.6 |
| MLP_72_80_32 | 2131 | 0.98 | 0.95 |  | 0.85 | 91.7/100.0/60.0/88.9 |
| MLP_258_128_64_do | 1437 | 0.74 | 0.83 |  | 0.85 | 83.3/90.0/90.0/77.8 |
| TinyResNet | 1686 | 0.73 | 0.93 |  | 0.78 | 83.3/100.0/70.0/55.6 |
| MLP_4_4_4 | 1190 | 0.80 | 0.77 |  | 0.73 | 83.3/100.0/40.0/66.7 |
| Nitta_et_al_6layer_linact | 228 | 0.48 | 0.80 |  | 0.34 | 100.0/0.0/30.0/22.2 |
| VGG_small_1 | 136 | 0.99 | 1.00 |  | 0.29 | 100.0/0.0/0.0/0.0 |
| VGG_small_3 | 811 | 0.90 | 1.00 |  | 0.29 | 100.0/0.0/0.0/0.0 |
| VGG_small_4 | 959 | 0.98 | 1.00 |  | 0.29 | 100.0/0.0/0.0/0.0 |
| MhNet1_bn_do_skipcon | 815 | 0.97 | 0.98 |  | 0.29 | 100.0/0.0/0.0/0.0 |
| VGG_small_2 | 122 | 0.99 | 1.00 |  | 0.24 | 0.0/0.0/100.0/0.0 |
| MhNet2_bn_do_skipcon | 162 | 0.86 | 0.90 |  | 0.24 | 0.0/100.0/0.0/0.0 |
| CNN_4conv2dense_optim | 271 | 0.92 | 0.98 |  | 0.24 | 0.0/0.0/100.0/0.0 |
| Nitta_et_al_6layer | - | - | - |  | - | - |
| Nitta_et_al_6layer_reluact | - | - | - |  | - | - |

^1^Further information regarding the neural network architectures is available at (5).

______

(5) Kräter M, Abuhattum S, Soteriou D, Jacobi A, Krüger T, Guck J, et al. AIDeveloper: deep learning image classification in life science and beyond. bioRxiv 2020;2020.03.03.975250.
